# LCA/LCC analysis of starting-lighting-ignition lead-acid battery in China

**DOI:** 10.7717/peerj.5238

**Published:** 2018-07-26

**Authors:** Yongxi Ma, Shuao Yu, Juanli Wang, Wei Yu

**Affiliations:** 1 School of Economics and Management, Zhejiang Sci-Tech University, Hangzhou, China; 2 Ecological Civilization Research Center of Zhejiang Province, Zhejiang Sci-Tech University, Hangzhou, China; 3 School of Management, Zhejiang University, Hangzhou, China; 4 School of Economics and Management, Zhejiang University of Water Resources and Electric Power, Hangzhou, China

**Keywords:** Life cycle costing, Life cycle assessment, Lead-acid battery, Environmental impact

## Abstract

**Background:**

China has the largest lead–acid battery (LAB) industry and market around the world, and this situation causes unavoidable emissions of Pb and other pollutants.

**Methods:**

On the basis of a field survey on a starting–lighting–ignition (SLI) LAB plant in Zhejiang Province, this study applies life cycle assessment (LCA) and life cycle costing (LCC) methods to assess the environmental impacts and environment-related costs derived from the LAB industry during the life phases, including material preparation, battery assembly, transportation, and regeneration of the plant.

**Results:**

Material preparation and regeneration phases contribute 3.4 and 42.2 g to Pb emission, respectively, and result in 3.29 × 10^8^ CHY of environmental cost for each function unit (1 KVA h LAB capacity). The material preparation phase is the largest mass contributor to global warming potential (GWP, 97%), photo-chemical oxidation potential (POCP, 88.9%), and eutrophication potential (EP, 82.5%) and produces 2.68 × 10^8^ CHY of environmental cost.

**Discussion:**

Decision makers in the Chinese LAB industry should replace the pyrogenic process in smelting with the use of clean energy, increase the lead recovery rate while producing the same capacity of LABs, and develop new technologies to reduce heavy metal emission, especially in the regeneration phase.

## Introduction

The lead–acid battery (LAB) is a broadly used power source around the world due to its apparent advantages, including low price, high unit voltage, stable performance, and capability to operate at extreme temperatures (Chang et al., 2009). According to a recent report from Grand View Research (2017), the market scale of LAB is expected to expand globally from 46.6 billion dollars in 2015 to 84.5 billion dollars in 2025 because of the environmental and sustainability issues caused by fossil energy consumption and a foreseeable increase in renewable energy storage systems in the manufacturing sector worldwide (Grand View Research, 2017). With its sharply increasing demand for electronic bikes, vehicles, and other systems using electronic power in the last decades, China currently holds the largest share of the global LAB market and accounted for 45% of the total global LAB output in 2015. Meanwhile, China generated 33.0 million tons of used LABs which contain 74% of lead plate for recycling with the potential profits of almost 62.0 billion CHY (9.8 billion U.S. dollars).

However, the LAB industry is associated with environmental and public health problems, especially the emission of lead, which is classified as one of the top heavy metal pollutants in China (Sun et al., 2017). An epidemiological investigation of 50 publicly reported lead poisoning cases in China from 2004–2012 (Lv, Kong & Rang, 2013) showed that 23 cases were related to LAB manufacturing and recycling and 19 were related to lead smelting. The amount of lead emission from the LAB industry reached a peak of 281 t in 2010 (Liu et al., 2017), and various lead poisoning scandals were reported at the same time (LaFraniere, 2011). From the view of life cycle management, the LAB industry is responsible for 84% of all lead poisoning cases, and batteries have become the most significant lead pollution source in China (Liu et al., 2015; Van der Kuijp, Huang & Cherry, 2013).

To reduce increasing lead emissions and hazards on public health, the Ministry of Environmental Protection of China (MEP) launched the “Cleaner Production Standard for Lead–Acid Battery Industry” in 2008 (MEP, 2008), “Cleaner Production Standard for Waste Lead–Acid Battery Recycling Industry” in 2009 (MEP, 2009), and “Technical Specifications of Pollution Control for Treatment of Lead–Acid Battery” in 2010 (MEP, 2010). Following the “12th Five-year Plan for Comprehensive Prevention of Heavy Metal Pollution” (MEP, 2011) enacted by China’s State Council, the MEP and eight other ministries jointly implemented a nationwide mandatory clean action to reduce heavy metal pollution in 2011. In this action, all LAB manufacturers and recyclers are required to suspend production until their environmental facilities are upgraded and pass a performance inspection by local environmental authorities. More than 80% of enterprises were either banned or suspended, and only 13% were qualified to operate (Liu et al., 2015). Moreover, the MEP and the Ministry of Industry and Information Technology (MIIT) of China issued “Access Conditions for Lead–Acid Battery Industry” and “Access Conditions for Secondary Lead Industry” in 2012, which clarified the detailed admittance requirements of environmental protection for LAB-related enterprises (MEP & MIIT, 2012a; MEP & MIIT, 2012b). Owing to these regulations, lead emissions from the LAB industry declined to 113 t in 2014 (Liu et al., 2017). Continually tackling lead pollution, MEP and MIIT also promulgated undated versions of the access conditions, namely, “Standard Conditions for Lead–Acid Battery Industry” in 2015 and “Policies for Pollution Control Techniques in Lead–Acid Battery Industry” in 2016 (MEP & MIIT, 2015; MEP, 2016). In 2017, “Extended Producer Responsibility (EPR)” was established by China’s State Council, which proposed a cradle-to-grave LAB monitoring system and placed the recycling responsibility on the manufacturer (CSC, 2017). After the change from end control mode to cleaner production, Chinese environmental management have entered the stage of whole process control.

Nonetheless, lead content loss remains common in the phases of Chinese LAB systems (Mao, Zhongwu & Yang, 2010). Decision makers in China have gradually recognized that emissions must be considered to achieve environmental sustainability. Key issues that could exert negative effects on the environment over the entire life cycle of LABs need to be assessed urgently. By considering impacts throughout the life cycle of a product, life cycle assessment (LCA) provides a comprehensive view of the environmental aspects of a product or process and a highly accurate picture of environmental trade-offs in product and process selection (Khasreen, Banfill & Menzies, 2009). This method has been proven to be useful and has been widely applied in studies on the environment and waste management (Abd Rashid et al., 2017; Finkbeiner et al., 2010; Ozkan et al., 2016; Rigamonti, Grosso & Giugliano, 2010; Singh et al., 2011). Several studies on LABs have focused on environmental performance and impacts from the perspective of product life cycle (Abd Rashid et al., 2017; Finkbeiner et al., 2010; Ozkan et al., 2016; Rigamonti, Grosso & Giugliano, 2010; Singh et al., 2011). Other studies have emphasized lead recycling and refining, the two procedures that produce the largest amount of pollution (Hong et al., 2017; Tian et al., 2017). Another branch of studies focused on traction batteries, which are mainly used in electric bicycles (Cherry, Weinert & Xinmiao, 2009; Ji et al., 2012; Liu et al., 2015; Wu, Ma & Li, 2015), calculation of emissions, and assessment of impacts in different phases.

Thus far, no specific studies have used LCA as a tool to assess the environmental impacts of starting–lighting–ignition (SLI) LABs. All studies mentioned were conducted without the life cycle costings (LCC) analysis integrated with LCA when studying LABs. The environmental impacts of SLI batteries need to be assessed because of the widespread use of SLI LABs in automobile internal combustion engines, motorcycles, and oversized vehicles and the market share of 25.6% in SLI batteries sales in the LAB output of China (Dong, 2013). The aim of this study is to assess the environmental impacts of SLI LABs in China by using the LCA method coupled with life cycle costing (LCC) analysis and present a clear view of the potential aspects that LAB manufacturers (or recyclers) should focus on to reduce negative environmental impacts. The findings from this study can provide provide input into further investigations on hazardous waste management to the government agencies and building professionals prior to future developments in terms of the environmental impact of LABs industry cycle in China.

The rest of the paper is organized as follows. ‘Methods’ presents the basic profile of the case under study, SLI LAB enterprises, and LCA/LCC methods, including function unit, research boundary, and life cycle phases. ‘Results and Discussion’ reports the results of life cycle impact assessment and life cycle environment costing. ‘Conclusions’ presents the conclusions, recommendations for enterprises, and policy proposals for the LAB industry.

## Methods

### LCA

This study follows the LCA technique standardised by ISO 14040 and 14044 instructions (ISO, 2006a; ISO, 2006b) which includes four steps: goal and scope definition, inventory analysis, impact assessment, and interpretation. Based on the economic input–output model developed by Leontief (Green Design Initiative, 2004), the LCA method coupled with life cycle costing (LCC) analysis is applied for the production and end-of-life stages of the product life cycle (Suh & Huppes, 2005). LCA/LCC analysis was carried out to develop a new economic method for internal (i.e., energy and material) and external cost (i.e., environmental) categories by using eBalance 4.0 software.

A typical LAB plant specializing in SLI batteries and located in Changxing County^1^ 1Changxing County, which contains more than half of LAB plants in China, is known as “the center of LABs.”in Zhejiang Province was selected for the case study and data acquisition. The database used in this study for LCA/LCC analysis was taken from on-site survey in the LAB production plant in China, academic literature and Chinese Life Cycle Database (CLCD). The primary data were obtained through a field survey on the LAB plant in 2016, which involved the enterprise reports (production, marketing, environmental monitoring/assessment, technical instruction, etc.). The necessary technical parameters were acquired from academic literature (Liu et al., 2015; Ozkan et al., 2016), LAB technical manuals (Pavlov, 2017) and so on. The CLCD database (IKE-global, 2014) which represented the Chinese market average technology was used for extracted the input–output inventory and environment impact assessment. The basic specifications of the SLI LABs in this study were as follows: energy/mass ratio of 36–44 W h/kg, energy/volume ratio of 80–120 W h/L, cell voltage rating of 12 V ×12 A h, service length of three years, and life charge cycle of 300–600 times.

#### Goal and scope definition

**Figure 1:**
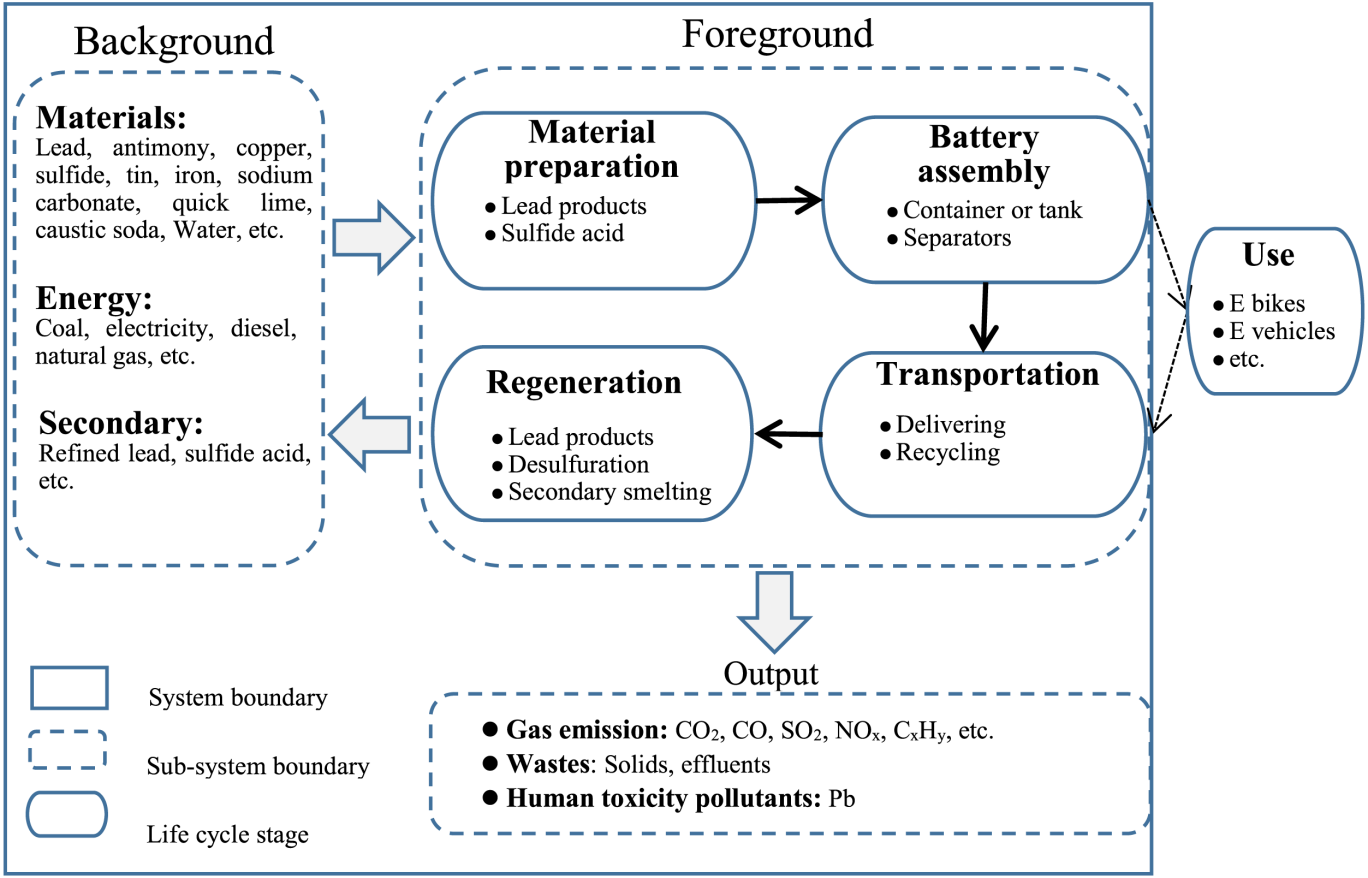
Life cycle of SLI Lead-Acid Batteries.

The main goal of this study is to evaluate the environmental impacts associated with the life cycle of SLI LABs. The function unit selected in this study was 1 KVA h LAB capacity to normalize data. The system boundary included three sub-system boundaries: foreground, background, and output sub-system(shown in Fig. 1). The foreground sub-system refers to the set of direct life cycle phases, including material preparation, battery assembly, transportation, and regeneration. The background sub-system considers the demand for assistive materials and energies in production and regeneration, and the output sub-system covers the pollutants derived from the entire system. The system boundary was set via a cradle-to-grave life cycle approach, and the phase of usage was not included for two reasons. First, SLI batteries generate less environmental burden than traction batteries do. Second, the LCA analysis focuses on a specific plant.

#### Life cycle inventory

Life cycle inventory (LCI) is a crucial part of LCA according to ISO 14040 standards. LCI clearly indicates inputs (e.g., materials and energies), outputs, and environmental releases throughout all life cycle phases. Specific inventory data were obtained from the involved LAB producer, on-site measurement, and information in literature. The data used for LCI were representative average data from secondary sources or data gathered from primary sources. The environmental impacts of 1 KVA h LAB capacity were separately shown in the life cycle phases (material preparation, battery assembly, transportation, and regeneration).

##### Phase 1: Material preparation.

This phase involves two sub-processes: plate making and paste mixing (including electrolyte). Lead–antimony alloy is the element used to make LAB plates, and pastes are composed of mixed water, sulfuric acid and lead powder. Pastes provide active materials in cell reaction, which directly determines the quality and electronic capacity of batteries. The LCI data of raw materials of plates and pastes are shown in Table 1.

**Table 1:** LCI data for producing 1 KVA h SLI LABs.

**Material**	**Input**		**Output**	
**Lead products: alloy, powder**	Lead (kg)	33.7	CO_2_(g)	2.56 × 10^5^
	Antimony (kg)	9.25	CO (g)	32
	Copper (kg)	2.45	SO_2_(g)	366
	Tin (kg)	0.19	NO_X_(g)	190
	Water (t)	0.01	C_X_H_Y_(g)	12
	Coal (kg)	97	Effluent (t)	1.56
	Electricity (kw h)	51	Solid (kg)	5.8
			C_X_H_Y_(g)	12
**Sulfuric acid**	SO_2_(kg)	56.85	CO_2_(g)	6.3 × 10^−4^
	Water (t)	0.03	CO (g)	20
	Coal (kg)	24	SO_2_(g)	79
	Electricity (kw h)	6	NO_X_(g)	57
			C_X_H_Y_(g)	5
			Effluent (t)	0.04

##### Phase 2: Battery assembly.

After material preparation, plates and pastes are ordered as positive/negative electrodes with battery separators and electrolytes and assembled into containers. The environmental impacts of generating separators and containers were not considered in this study because they are not directly produced in the case plant. According to primary data, assembling 1 KVA h of LABs costs 4 kw h of electricity.

##### Phase 3: Transportation.

The next phase is to deliver the assembled LABs to markets (e.g., dealers and franchisees). The usage phase of SLI LABs was not included in this study. Thus, after delivery, another part of transportation is to recycle spent LABs for regeneration. According to primary data from the field survey and enterprise reports, the average distance from the plant to the market/recycle point is 177 km. Considering two round-way trips in the case wherein the market and recycle point are not exactly located in the same place, the total transportation of a life cycle should be 708 km. Specifically, 5 t diesel trucks (diesel consumption: 28 L/100 km) execute the transportation. The LCI data of transportation are shown in Table 2.

**Table 2:** LCI data of transporting 1 KVA h SLI LABs.

Input		Output	
Diesel (L/km)	0.28	CO_2_ (g)	670
Distance (km)	708	CO (g)	2.5
Total diesel (L)	198.24	SO_2_(g)	13
		NO_X_(g)	45
		C_X_H_Y_(g)	1.5

##### Phase 4: Regeneration.

Spent LABs are commonly recognized as high-value resources for secondary lead industries. According to an investigation, 85% of recycled LABs are used to recover lead, with a high recovery rate of 92%. In this case, regeneration of spent SLI LABs involves three main process flows. First, in the crushing and separation process, lead pastes and granules are separated from the mixture of spent sulfuric acid, plastic, and rubber. Second, pretreatment, including purification of spent acid and desulfurization of mixtures, is applied to eliminate or at least mitigate the impact of pollutants derived from crushing and separation. Finally, spent lead pastes and granules are smelted to refine secondary lead. Table 3 provides the inventory data of regenerating 1 KVA h secondary SLI LABs.

**Table 3:** LCI data of regenerating 1 KVA h secondary SLI LABs.

	**Input**		**Output**	
**Crushing and separation**	Water (t)	0.08	SO_2_(g)	7.5
	Electricity (kw h)	1	Effluent(t)	0.03
			Solid(kg)	1.25
			Pb (g)	0.2
**Pretreatment**	Sodium carbonate carbonate (kg) (kg)	0.88	CO_2_(g)	132.5
	Water (t)	0.04	SO_2_(g)	63.8
	Coal (kg)	0.5	NO_X_(g)	2.4
	Electricity (kw h)	6	C_X_H_Y_(g)	2.5
			Pb (g)	13
			Effluent(t)	0.018
			Solid(kg)	0.23
**Secondary lead smelting**	Sodium carbonate (kg)	0.06	CO_2_(g)	5,882
	Caustic soda (kg)	0.08	SO_2_(g)	23.2
	Quick lime (kg)	0.04	NO_X_(g)	5
	Iron scurf (kg)	0.4	C_X_H_Y_(g)	5
	Water (t)	0.06	Pb (g)	29
	Coal (kg)	1.7	Effluent (t)	0.032
	Electricity (kw h)	22.5	Solid (kg)	0.52
	Natural gas (m^3^)	2.7		

#### Life cycle impact assessment (LCIA)

Three impact categories (including common impacts, waste and toxicity, and resource depletion) were selected to assess the environmental loads of LAB. The three impact categories were further divided into 10 sub-categories. The common impact category covers four common environmental consequences of human activities, which are global warming potential (GWP), acidification potential (AP), photo-chemical oxidation potential (PCOP), and eutrophication potential (EP). Next, considering the waste and toxicity impact of the LAB industry, an LAB plant may create waste solids and water with heavy metal pollutants (especially Pb) in them. In this category, solid waste (SW), effluent (EF), and heavy metal emission (HME) are set as sub-categories. Resource depletion potentials are classified as the third category, where water depletion potential (WDP), abiotic resource depletion potential (ADP), and energy depletion potential (EDP) are included (Goedkoop et al., 2009; Hauschild et al., 2011). All categories of LCIA, including the normalization standards of the common impact category, are shown in Table 4.

**Table 4:** LCIA categories of LABs.

**Category**	**Sub-Category**	**Elements**	**Normalization**	
**Common impact**	GWP	CO_2_, CO	CO_2_	1g CO–2g CO_2_
	AP	SO_2_, NO_x_	SO_2_	1g NO_x_–0.7g SO_2_
	POCP	C_x_H_y_	C_2_H_2_	1g C_x_H_y_–0.4g C_2_H_2_
	EP	NO_x_	NO_3^−^_	1g NO_x_–1.35g NO_3^−^_
**Waste and toxicity**	SW	Solid wastes	–	–
	EF	Effluents	–	–
	HME	Pb	–	–
**Resource depletion**	WDP	H_2_O	–	–
	ADP	Pb, Sb, NaCO_3_, CaCO_3_, NaOH_2_	–	–
	EDP	Coal, electricity, diesel, gas	–	–

### LCC

LCC in this study focused on environmental costs in the life cycle of SLI LABs. The LCC analysis in this part covered three environmental cost categories corresponding to LCIA: common impact cost, waste and toxicity cost, and resource depletion cost. Common impact cost measures the potential economic or social losses caused by global warming, acidification potential, photo-chemical oxidation, and eutrophication (Goedkoop et al., 2009; Hauschild et al., 2011). Waste and toxicity cost refers to: (1) the fees involved in dealing with solid wastes and effluents and (2) hazards to human health induced by heavy metal pollutants. Resource depletion cost covers the costs of input materials and energies. The unit costs or prices of LCIA items derived from the primary survey and relevant studies are listed in Table 5.

**Table 5:** Unit costs of LCIA categories for SLI LABs.

**Common impact**	**Unit cost**	**Resource depletion**	**Unit cost**
GWP^1^	930 CHY/g	H_2_O (WDP)^2^	3.15 CHY/t
AP^1^	6.26 × 10^4^ CHY/g	Pb (ADP)^3^	17.8 CHY/kg
POCP^1^	1.63 × 10^4^ CHY/g	Sb (ADP)^3^	44 CHY/kg
EP^1^	2.97 × 10^4^ CHY/g	Cu (ADP)^3^	45 CHY/kg
		Sn (ADP)^3^	140 CHY/kg
Waste and toxicity	Unit cost	Fe (ADP)^3^	2.7 CHY/kg
SW^3^	180 CHY/t	CaCO_3_(ADP)^3^	0.3 CHY/kg
EF^2^	2.45 CHY/t	NaCO_3_(ADP)^3^	1.5 CHY/kg
HME^1^	7.22 × 10^6^ CHY/g	NaOH_2_(ADP)^3^	2.5 CHY/kg
		Coal (EDP)^3^	0.8 CHY/kg
		Electricity (EDP)^2^	0.67 CHY/kw h
		Diesel (EDP)^2^	5.5 CHY/L
		Natural gas (EDP)^2^	4.8 CHY/m^3^

## Results and Discussion

### LCIA

**Figure 2:**
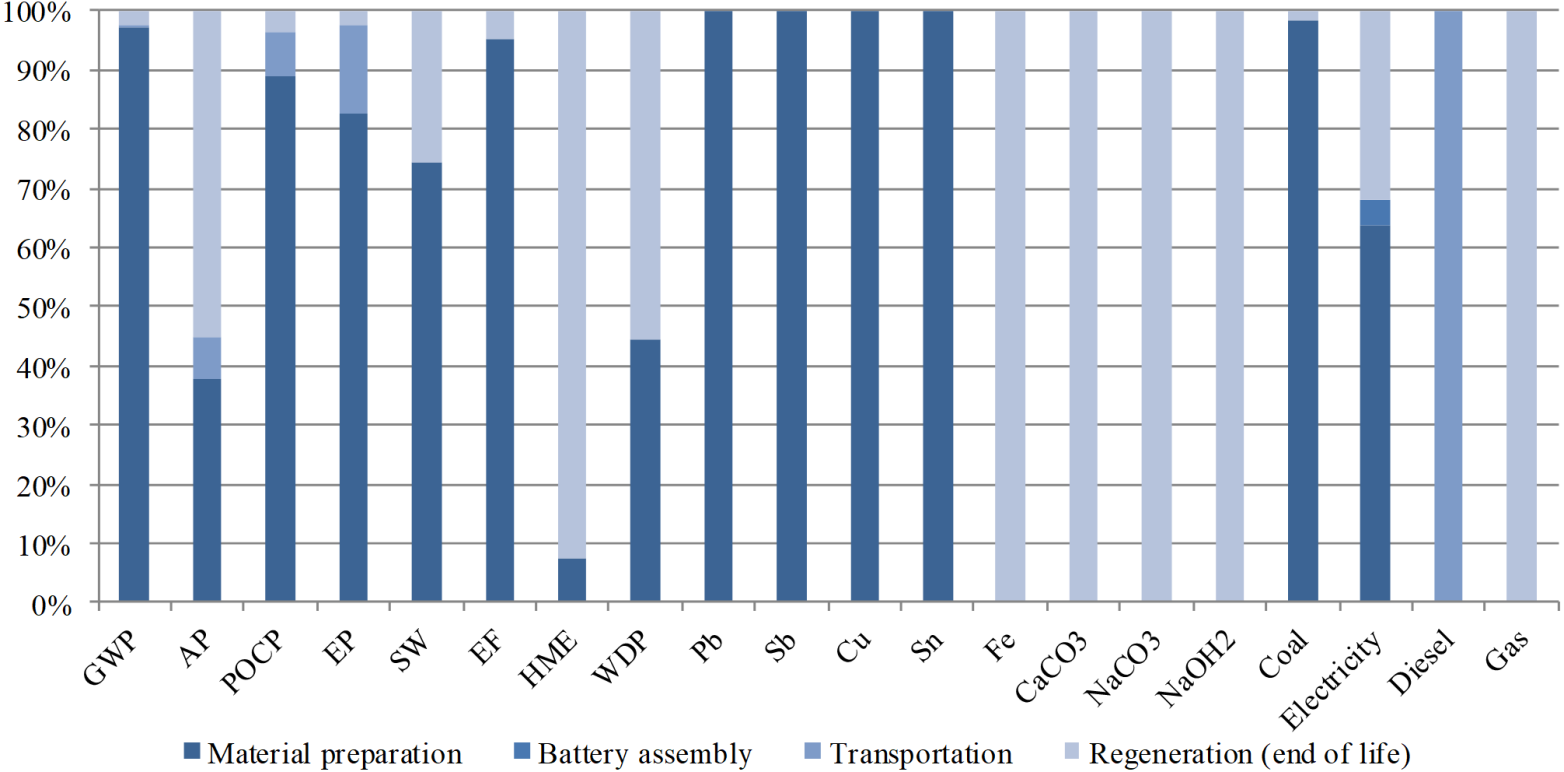
Environment impact distribution in the life cycle phases. Shows the environment impact distribution in the life cycle phases. The material preparation and regeneration phases affect the environment heavily during the life cycle of LAB production. GWP, HME, AP, and EP are the main effects. The material preparation phase has the highest level of GWP due to the use of fossil fuel, and the regeneration phase has the highest HME impact because of the improper management of lead smelting.

Table 6 summarizes the results of LCIA for 1 KVA h LAB capacity calculated at the midpoint level. Figure 2 shows the environment impact distribution in the life cycle phases. The material preparation and regeneration phases affect the environment heavily during the life cycle of LAB production. GWP, HME, AP, and EP are the main effects. The material preparation phase has the highest level of GWP due to the use of fossil fuel, and the regeneration phase has the highest HME impact because of the improper management of lead smelting.

**Table 6:** LCIA results of 1 KVA h SLI LABs.

**Category**	**Sub-category**	**Unit**	**Entire life cycle**	**Material preparation**	**Battery assembly**	**Transportation**	**Regeneration (end of life)**
Common impact	GWP	g CO_2_-eq	2.63 × 10^5^	2.56 × 10^5^	–	675	6014.5
	AP	g SO_2_-eq	180.7	68	–	13	99.7
	POCP	g C_2_H_2_-eq	8.1	7.2	–	0.6	0.3
	EP	g NO_3^−^_-eq	404.3	333.5	–	60.8	10
Waste and Toxicity	SW	kg	7.8	5.8	–	–	2
	EF	t	0.168	0.16	–	–	0.008
	HME	g	45.6	3.4	–	–	42.2
Resource depletion	WDP	t	0.09	0.04	–	–	0.18
	ADP						
	Pb	kg	33.7	33.7	–	–	–
	Sb	kg	9.25	9.25	–	–	–
	Cu	kg	2.45	2.45	–	–	–
	Sn	kg	1.75	1.75	–	–	–
	Fe	kg	0.4	-	–	–	0.4
	CaCO_3_	kg	0.04	-	–	–	0.04
	NaCO_3_	kg	0.94	-	–	–	0.94
	NaOH_2_	kg	0.08	-	–	–	0.08
	EDP						
	Coal	kg	123.2	121	–	–	2.2
	Electricity	kw h	87.5	57	4	–	28.5
	Diesel	L	49.7	-	–	49.7	
	Gas	m^3^	2.7	-	–	–	2.7

LABs themselves do not create gas pollutants that cause common environmental impacts, but the uses of energy uses during the LAB life cycle, such as coal burning, generate elements that are likely to induce GWP (CO_2_), AP (SO_2_), PCOP (*C*_*x*_*H*_*y*_), and EP (NO_*x*_). The entire life cycle of 1 KVA h LAB capacity indicates that 2.63 × 10^5^ g of the GWP element CO_2_ is released to the atmosphere. Owing to the high coal consumption portion (98.2%) among phases, 97% of the total CO_2_ emission is generated from the material preparation phase, in which primary lead and other metals are smelted to form alloy and other lead products. For the same reason, material preparation releases a large amount of PCOP (88.9%) and EP (82.5%) pollutants as well. Meanwhile, diesel burning from the transportation phase generates 0.7% of GWP, 7.2% of AP, 7.4% of PCOP, and 15% of EP. The end of life phase, regeneration, consumes limited coal (1.8%) but contributes more than half to the total life cycle AP pollutant (55.2%) because in this phase, desulfuration of spent LABs is one of the main purposes.

Similar to that in the common impact category, the material preparation phase generates the major portion of wastes, namely, 74.4% of total solid wastes and 95.2% of total effluents. Moreover, 3.4 g of toxic heavy metal is released to environment along with these wastes. Poisonous heavy metal (42.2 g) emission also occurs in the regeneration phase, and these poisonous heavy metals account for 92.5% of the total heavy metal emission in the entire life cycle. Processes, such as spent LAB crushing, desulfuration, and secondary lead smelting, are all potential heavy metal emission sources.

Resource depletion is also affected significantly by material preparation and regeneration. Producing 1 KVA h LAB capacity requires 0.04 t of freshwater, 33.7 kg of lead, 9.25 kg of antimony, 2.45 kg of copper, and 1.75 kg of tin. In regeneration processes, 0.4 kg of iron, 0.94 kg of sodium carbonate, 0.08 kg of caustic soda, and 0.04 kg of quick lime are inputted to spent LAB desulfuration and secondary lead smelting. With regard to the primary energy use of 1 KVA h LAB capacity, material preparation consumes 98.2% of the total coal use and 65.1% of the total electricity use, battery assembly expends 4 kw h of electricity (4.6% of the total use), and delivery and recycling require 49.7 L of diesel for transportation. In the EOL phase, 1.8% of total coal use, 32.6% of total electricity use, and 2.7 m^3^ of natural gas are consumed.

### LCC

The results of LCC for 1 KVA h LAB capacity are given in Table 7. Overall, the life cycle costings of “common impact” and “waste and toxicity” result in a large amount of potential economic loss (268 and 329 million CHY, respectively) for the environment. GWP and HME account for the major environmental costs, and their costs exceed those of the other impact sub-categories. The total environmental costs are mainly shared by the material preparation and regeneration phases, with percentages of 55% and 45%, respectively.

**Table 7:** LCC results of 1 KVA h SLI LABs (unit: CHY).

**Category**	**Sub-category**	**Whole life cycle**	**Material preparation**	**Battery assembly**	**Transportation**	**Regeneration (end of life)**
**Common**	GWP	2.45 × 10^8^	2.38 × 10^8^	0	6.28 × 10^5^	5.59 × 10^6^
**Impact**	AP	1.13 × 10^7^	4.26 × 10^6^	0	8.14 × 10^5^	6.24 × 10^6^
	POCP	1.32 × 10^5^	1.17 × 10^5^	0	9.78 × 10^3^	4.89 × 10^3^
	EP	1.20 × 10^7^	9.90 × 10^6^	0	1.81 × 10^6^	2.97 × 10^5^
	**sum**	2.68 × 10^8^	2.52 × 10^8^	0	3.26 × 10^6^	1.21 × 10^7^
**Waste and**	SW	1.40	1.04	0	0	0.36
**Toxicity**	EF	0.41	0.39	0	0	0.02
	HME	3.29 × 10^8^	2.45 × 10^7^	0	0	3.05 × 10^8^
	**sum**	3.29 × 10^8^	2.45 × 10^7^	0	0	3.05 × 10^8^
**Resource**	WDP	0.29	0.13	0	0	0.16
**depletion**	ADP	1,363.5	1,362.2	0	0	1.38
	EDP	443.5	135	2.68	273.4	33.76
	**sum**	1,807.3	1,497.3	2.68 (0.2%)	273.4 (15.1%)	35.30

**Figure 3:**
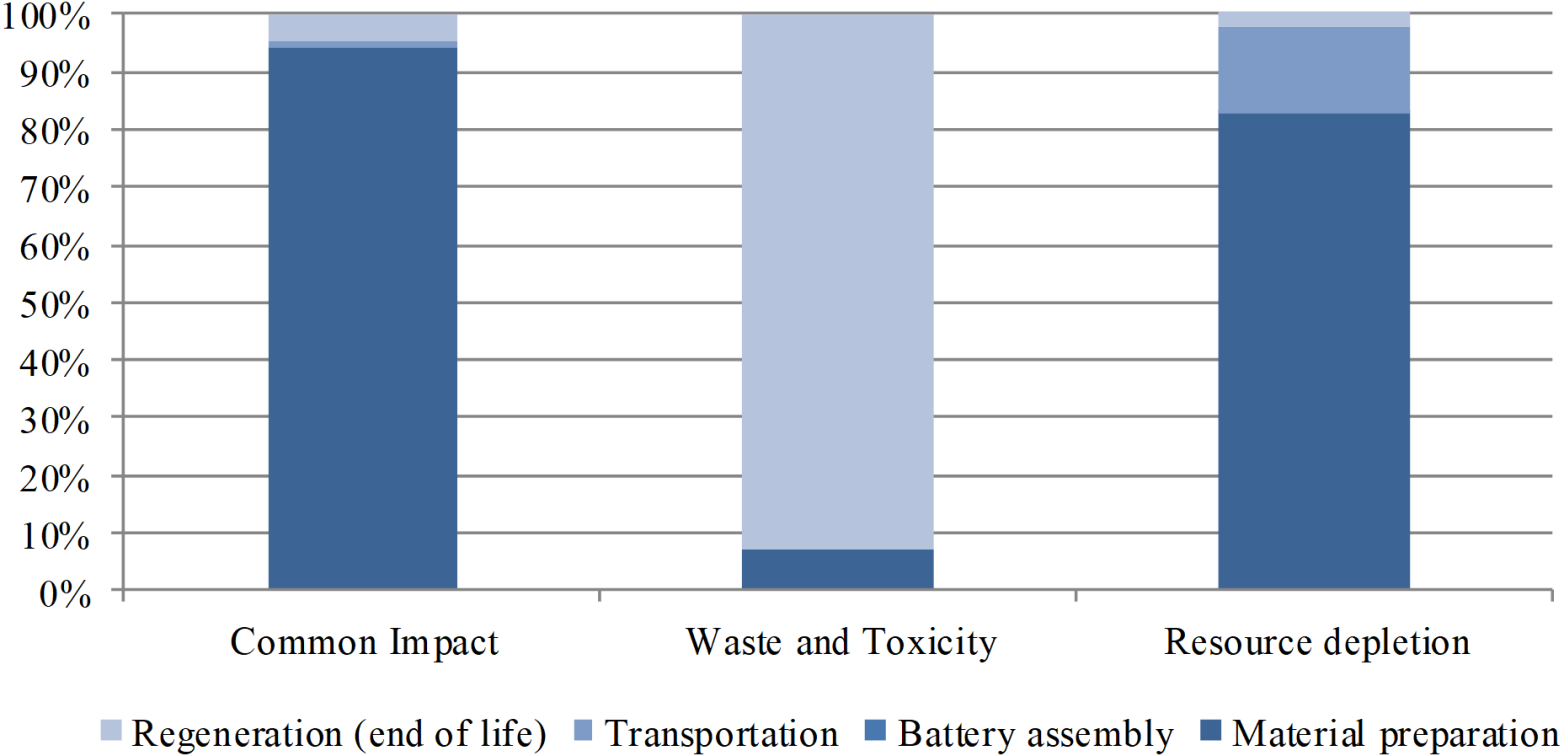
Cost source distribution in the life cycle phases. For common impact, the potential economic loss is mainly derived from GWP. During the life cycle, 94.3% of the total cost in this category originates from the material preparation phase, 4.5% from the regeneration phase, and 1.2% from the transportation phase. The regeneration phase accounts for 92.7% of the total cost due to its major role in Pb emission during the life cycle. The material preparation phase accounts for 82.8% of the total cost during the life cycle because most resources and energies, especially abiotic resources, are inputted in this phase.

The costings source distribution in the life cycle phases is shown in Fig. 3. For common impact, the potential economic loss is mainly derived from GWP. During the life cycle, 94.3% of the total costing in this category originates from the material preparation phase, 4.5% from the regeneration phase, and 1.2% from the transportation phase. The regeneration phase accounts for 92.7% of the total costing due to its major role in Pb emission during the life cycle. The material preparation phase accounts for 82.8% of the total costing during the life cycle because most resources and energies, especially abiotic resources, are inputted in this phase.

**Figure 4:**
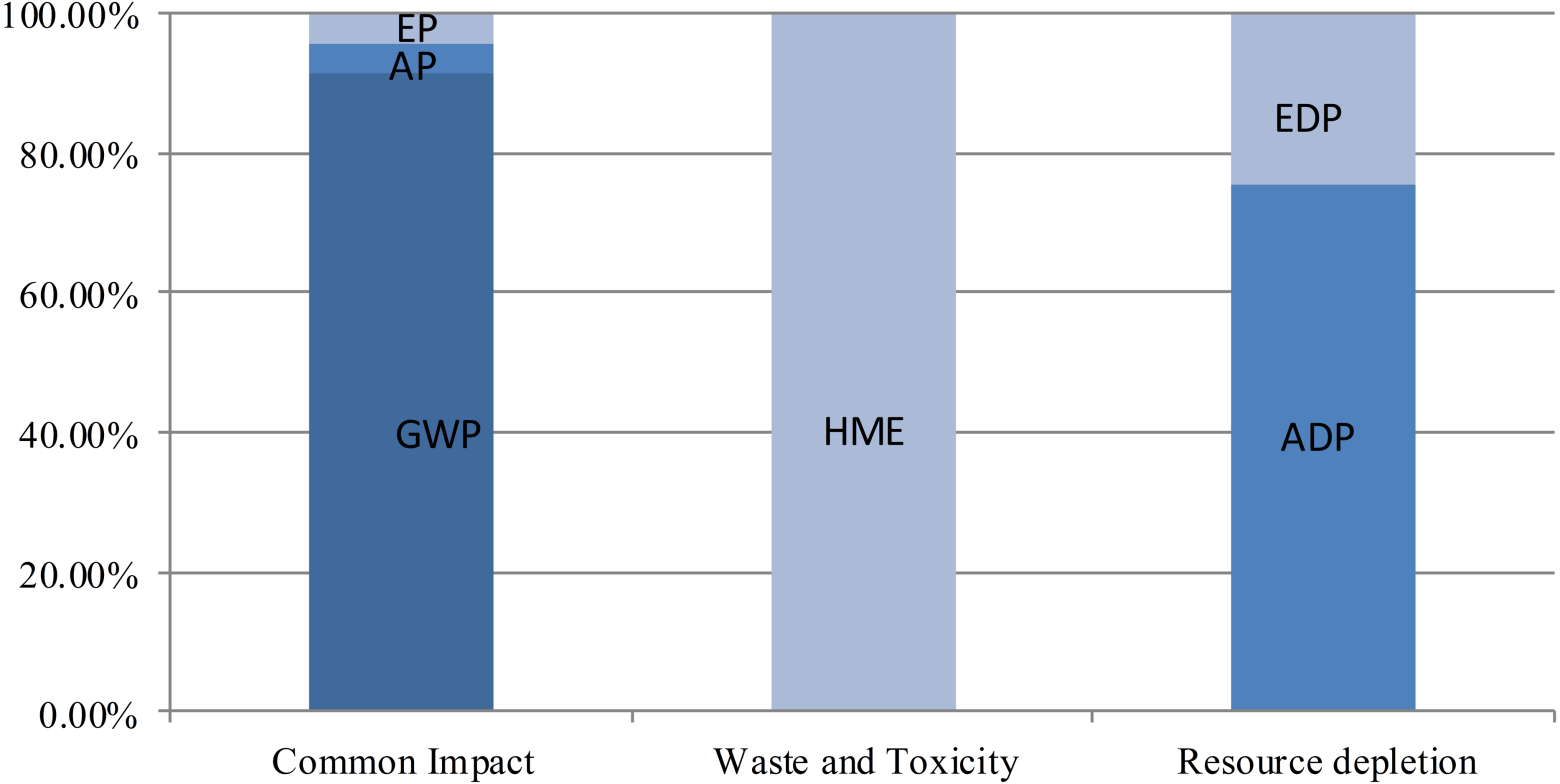
Sub-category costings distribution in the three environment impact categories. GWP-induced loss accounts for 91.3% of the total costing, followed by 4.5% from EP and 4.2% from AP. Although POCP results in 1.32 × 10^5^ CHY of cost, the value is only equal to slightly over 1% of the cost from AP (1.20  × 10^7^ CHY). HME accounts for the major proportion of potential economic loss in the waste and toxicity category. Compared with the fees for dealing with SW (1.4 CHY) and EF (0.41 CHY) in the life cycle, the human health risk costs (3.29  × 10^8^ CHY) induced by HME (Pb) is millions of times higher. In the resource depletion category, EDP cost accounts for a quarter of the total costing, and ADP contributes three-fourths. With a very low price of water (3.15 CHY/t), WDP is merely 0.49 CHY in this part. The rest of the costs in this category from the other three phases of the life cycle are mainly from EDP.

The sub-category costings distribution in the three environment impact category is shown in Fig. 4. Among the sub-categories of common impact, GWP-induced loss accounts for 91.3% of the total costing, followed by 4.5% from EP and 4.2% from AP. Although POCP results in 1.32 × 10^5^ CHY of costing, the value is only equal to slightly over 1% of the costing from AP (1.20 × 10^7^ CHY). HME accounts for the major proportion of potential economic loss in the waste and toxicity category. Compared with the fees for dealing with SW (1.4 CHY) and EF (0.41 CHY) in the life cycle, the human health risk costing (3.29 × 10^8^ CHY) induced by HME (Pb) is millions of times higher. In the resource depletion category, EDP cost accounts for a quarter of the total cost, and ADP contributes three-fourths. With a very low price of water (3.15 CHY/t), WDP is merely 0.49 CHY in this part. The rest of the costings in this category from the other three phases of the life cycle are mainly from EDP.

### Sensitivity analysis

**Figure 5:**
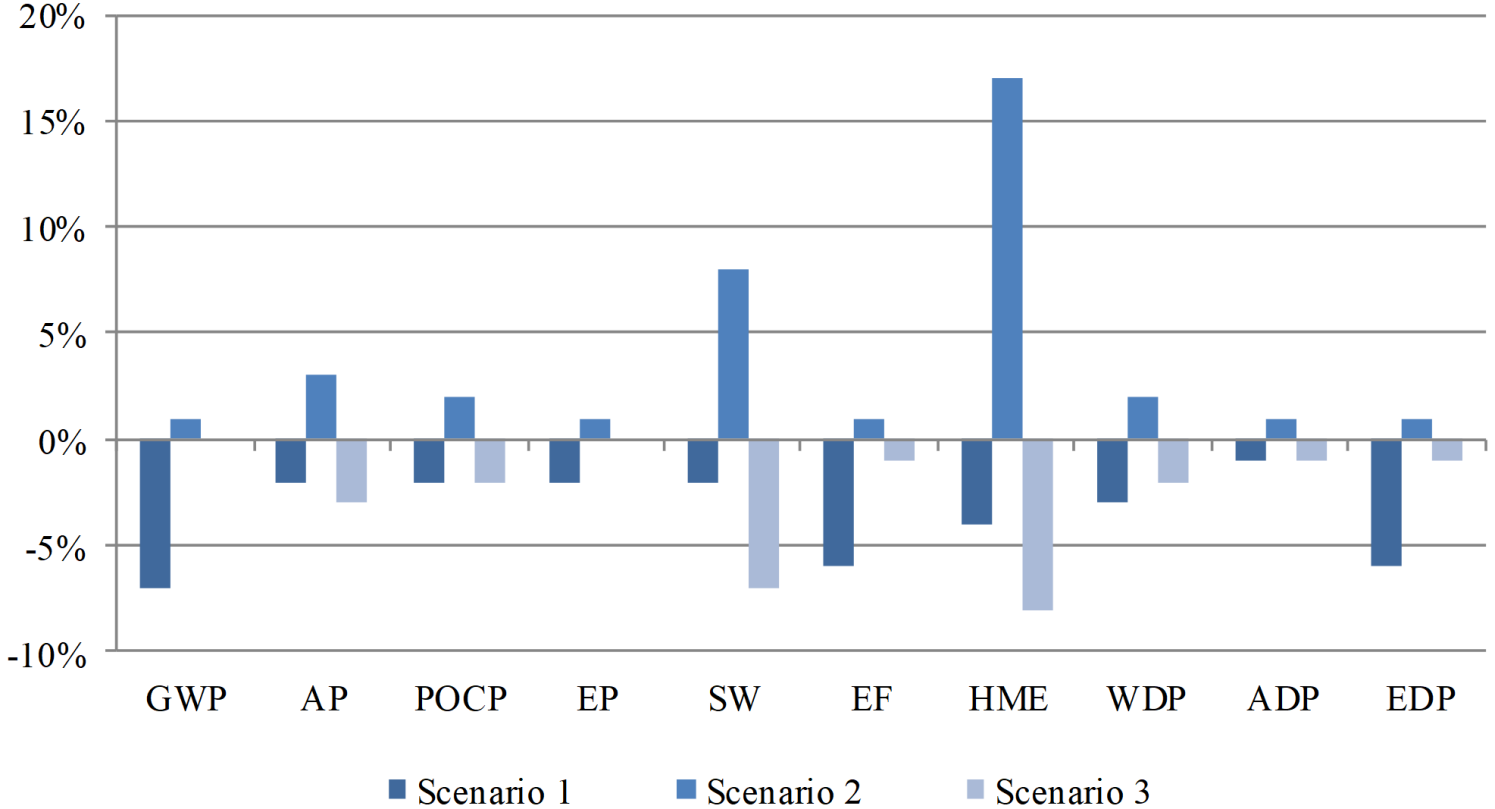
Sensitivity analysis results of energy input and lead recovery. The variation in energy input in the material preparation phase exerts a large impact on GWP, and HME is highly sensitive to the lead recovery rate.

A sensitivity analysis was performed to evaluate the reliability of the results. The sensitivity coefficients were employed to evaluate the reliability of the above results. The LCIA results indicate that energy input and lead recovery are crucial factors that influence environmental performance. Then, three different scenarios were set for the sensitivity analysis: (1) the energy input was decreased by 5% in the material preparation phase; (2) a recovery rate of 88% was set for lead in the regeneration phase; and (3) a recovery rate of 96% was set for lead in the regeneration phase. The scenario analysis results are shown in Fig. 5. The sensitivity analysis demonstrated that the variation in energy input in the material preparation phase exerts a large impact on GWP, and HME is highly sensitive to the lead recovery rate.

### Discussion

LCA and LCC analysis on SLI LABs from a systematic perspective in this study have a high potential for moving industrial practice towards sustainable development. An LCC analysis integrated with LCA, taking into account long-term costs and environmental effects, may contribute to more sustainable decision-making for administrative departments to develop cleaner production and environment management in LABs industry.

In view of the results mentioned above, several improvement measures can be adopted to reduce the environmental impacts in all impact categories and to reduce potential costs for the LAB industry in China. Fossil fuel (especially coal) burning is responsible for the common impact on the environment. Gas emissions strongly correlated to GWP, AP, POCP, and EP are mainly released during the two coal-consuming phases: during material preparation when primary lead products are processed and during regeneration when secondary lead is smelted. Replacing coal in these two phases with clean energy, such as natural gas and electricity (generated by renewable energy such as solar and wind), may effectively reduce gas emissions and future environment costs (potential economic loss).

According to results of LCIA/LCC, although 33.7 kg of Pb consumed in producing 1 KVA h LAB capacity, there is still 45.6 g of Pb released to environment. Given that Pb is a poisonous element for humans, emission of this heavy metal poses a serious risk to public health. Although alternative choices, such as lithium batteries, involve fewer HME issues, their high costs and storage problems make the widespread use of these alternatives impractical. As recycling materials to produce new batteries can decrease the lead pollution, the secondary lead industry should be supported and get opportunities to develop. Considering lead recovery rate had a highly effect on HME, the secondary lead enterprises with low recovery rate should be banned under strict supervision for environmental protection.

Therefore, the LAB industry in China should focus on a means to utilize less Pb while generating the same capacity of LABs and must control Pb emission from the very beginning. Although small plants with improper management in China have disappeared recently due to strict sequential regulations, most existing LAB entrepreneurs still use a pyrogenic process to refine and smelt lead and other metals. This refine method is effective, flexible, and inexpensive, but it results in high environment loads because of the burning of large amounts of coal. New methods characterized by reduced pollution and emission should be developed and applied in the LAB industry to reduce the potential loss induced by common environmental impacts and HME-related toxicity issues.

## Conclusions

This study applied LCA and LCC methods to an SLI LAB plant in China, and three life cycle impact categories (including 10 sub-categories) were assessed with their environment costings from cradle-to-grave life cycle phases. For the common impact category, material preparation is the largest mass contributor to GWP (97%), POCP (88.9%), and EP (82.5%) and accounts for 94% of the total life cycle costing in this category. Material preparation and regeneration phases release 3.4 and 42.2 g of heavy metal, respectively, with regard to the wastes and toxicity category because of the lead smelting procedures in these two production phases; they result in 3.29 ×10^8^ CHY of environmental cost induced by the human health risk posed by poisonous pollutant from heavy metal. The material preparation phase also consumes a large portion of ADP and EDP and contributes 82.8% to the total costing of the resource depletion category. Overall, the results show that the material preparation and regeneration phases of LAB life cycle create GWP, AP, POCP, and EP issues because of coal burning and other resource depletion processes. The regeneration phase also results in serious heavy metal emission during crushing, pretreatment, and re-smelting processes. To meet the growing requirements of and address pressures on LABs in China, the study suggests that LAB plants replace the pyrogenic process in smelting by using clean energy, increase the lead recovery rate while producing the same capacity of LABs, and develop new technologies to reduce Pb emission, especially in the generation phase.

## Additional Information and Declarations

### Data Availability

The following information was supplied regarding data availability: The raw data are included in the Tables.
